# Production of Porous Agarose-Based Structures: Freeze-Drying vs. Supercritical CO_2_ Drying

**DOI:** 10.3390/gels7040198

**Published:** 2021-11-05

**Authors:** Mariangela Guastaferro, Lucia Baldino, Ernesto Reverchon, Stefano Cardea

**Affiliations:** Department of Industrial Engineering, University of Salerno, Via Giovanni Paolo II, 132, 84084 Fisciano, Italy; mguastaferro@unisa.it (M.G.); ereverchon@unisa.it (E.R.); scardea@unisa.it (S.C.)

**Keywords:** agarose, aerogel, cryogel, supercritical CO_2_ drying, freeze-drying, biopolymer, scaffold, tissue engineering

## Abstract

In this work, the effect of two processes, i.e., freeze-drying and supercritical CO_2_ (SC-CO_2_) drying, on the final morphology of agarose-based porous structures, was investigated. The agarose concentration in water was varied from 1 wt% up to 8 wt%. Agarose cryogels were prepared by freeze-drying using two cooling rates: 2.5 °C/min and 0.1 °C/min. A more uniform macroporous structure and a decrease in average pore size were achieved when a fast cooling rate was adopted. When a slower cooling rate was performed instead, cryogels were characterized by a macroporous and heterogenous structure at all of the values of the biopolymer concentration investigated. SC-CO_2_ drying led to the production of aerogels characterized by a mesoporous structure, with a specific surface area up to 170 m^2^/g. Moreover, agarose-based aerogels were solvent-free, and no thermal changes were detected in the samples after processing.

## 1. Introduction

Polysaccharides, such as alginate, agarose and chitosan, are carbohydrate polymers that exhibit appropriate physicochemical features for biomedical applications and for controlled drug delivery [1,2,3,4]. In particular, agarose, together with its derivatives and blends, is widely used in tissue engineering and regenerative medicine due to its pH-responsive properties, resemblance to the extracellular matrix, controllable permeation for nutrients, inert structure, thermo-reversible gelation behavior and poroelastic structure [5,6,7,8]. Moreover, agarose biomechanical properties can be modulated in a wide range of ways, depending on the kind of soft or hard tissue to substitute and by varying the polymer concentration [9,10]. For example, low concentrations of agarose are able to reproduce the dynamic porous media behavior of soft tissues (i.e., brain) with adequate water absorption and retention properties [11,12]. On the other hand, Fey et al. [13] prepared different mineral foams consisting of agarose, alumina, hydroxyapatite and calcium phosphate that could be used for hard tissue applications.

Agarose-based scaffolds are generally obtained from hydrogels. Hydrogel formation starts by dissolving agarose in hot water at 80–90 °C. At temperatures higher than 37 °C, agarose immersed into the aqueous system remains in the sol-state and exists as a disordered ‘random coil’. Upon solution cooling, the Brownian diffusion of the polymeric chains slows down and the agarose-based hydrogel adopts an ordered double helix state [14,15,16].

Applications to which agarose may be addressed often need dried samples for preservation, transport and storage. For this purpose, different drying techniques have been investigated, such as air drying, freeze-drying and supercritical CO_2_ (SC-CO_2_) drying [10,17,18]. The dried solid materials can be termed as ‘cryogels’ when water inside the hydrogel is sublimated by freeze-drying, whereas ‘aerogels’ are produced by SC-CO_2_ drying. Ramya et al. [17] synthesized agarose-gelatine-hydroxyapatite composite cryogels for skin regeneration. In order to dry the gels, these authors used a combination of freeze-drying with gamma irradiation at various dosages, i.e., 25 KGv, 50 KGv and 100 KGv. Gamma irradiation was used to promote pore formation on the scaffold surface, as a direct consequence of an induced localized heating. However, closed pores were obtained, and, after irradiation, a drastic reduction in mechanical properties was also recorded. Luo et al. [10] realized an osteoconductive tricomposite cryogel of N-graphene (NG)/hydroxyapatite (HAp) hybrids blended with an agarose matrix via hydrothermal/cross-linking/freeze-drying. The introduction of NG and HAp significantly improved the gel’s mechanical properties, thus promoting the proliferation and viability of mesenchymal stem cells (MSCs). Kazimierczac et al. [19] realized chitosan–agarose–hydroxyapatite scaffolds combining gas foaming and freeze-drying. Sivashankari et al. [20] investigated the degradation kinetics of porous scaffolds based on agarose and chitosan. Graphene oxide was added to avoid fast scaffold degradation, to offer a higher mechanical stability for long term applications and to improve cell proliferation. The average pore size of agarose–chitosan-based scaffolds was 249 ± 35 µm; whereas, by increasing graphene oxide concentrations, the average pore size increased up to 306 ± 46 µm. Yuan et al. [21] produced a polymeric blend using agarose and konjac glucomannan for ciprofloxacin release. Freeze-drying was used as the drying technique. At the end of the process, cryogels were characterized by an almost completely closed and irregular structure. Moreover, a significant burst effect of the drug was recorded; i.e., more than 95% of ciprofloxacin was released in the first half hour, since most of it was not efficiently encapsulated into the polymeric structure.

Summarizing the results obtained from the previous works, during the freeze-drying process, the hydrogels are firstly frozen and, then, sublimation of the solvent (water) is achieved at a low pressure, avoiding the formation of vapor–liquid meniscus. In this way, the macroscopic gel structure can be preserved; however, the resulting morphology is generally characterized by macropores and brittle and fragile behavior [17,20,21].

In the biomedical field, gels should present specific features, such as: high porosity, large internal surface, tunable and regular average pore diameter and interconnected 3-D structure [3,22,23,24]. Moreover, gels should be characterized by a mesoporous structure, since this kind of morphology is similar to the native tissue extracellular matrix and, subsequently, can enhance cell adhesion and growth [25,26,27]. Therefore, a crucial step is to preserve the open interconnected nanostructure of the native hydrogel after drying. SC-CO_2_ gel drying seems to be the most appropriate technique to achieve this goal [28,29,30]. Tabernero et al. [30] produced nanostructured alginate–gelatin aerogels by SC-CO_2_ drying. This process was able to maintain the native nanoporous gel structure and, by varying the pressure and temperature conditions of the process, the porosity values and aerogel morphology were tuned. Baldino et al. [29] realized nanoporous ampicillin loaded alginate aerogels to provide a controlled and sustained drug release. The same authors [28] performed the supercritical drying process of chitosan–gelatin mixtures in order to produce aerogels with enhanced mechanical properties. The nanoporous structure obtained, combined with a Young’s modulus larger than 150 KPa of the samples, made these aerogels suitable for bone regeneration. It is worth noting that SC-CO_2_ drying is able to preserve the delicate wet gel nanoporous structure when the process is carried out at supercritical conditions of the mixture organic solvent + CO_2_, and the vapor–liquid meniscus is not formed. In this way, it is possible to realize aerogels that are suitable as scaffolds for tissue regeneration thanks to the mesoporous open pore network, high surface area and porosity [30,31,32,33]. Witzler et al. [18] investigated the release behavior of agarose–hydroxyapatite-based scaffolds that were obtained using two different techniques: freeze-drying and SC-CO_2_ drying. Freeze-dried samples exhibited large pores in the range of several hundred micrometers. This result could be attributed to the slow freezing process, which led to a formation of large ice crystals where, during sublimation, these crystals induced the formation of a macroporous structure. In contrast, samples that were supercritically dried exhibited a nanoporous and homogenous structure. In addition, Baudron et al. [34] performed a morphological comparison between starch porous materials obtained via SC-CO_2_ drying and freeze-drying, and demonstrated that aerogels were superior than cryogels in terms of their nanostructure and surface area (up to about 200 m^2^/g).

Therefore, the scope of this work is to systematically compare the morphology of agarose-based structures produced using two different processes, freeze-drying and supercritical CO_2_ drying, in order to determine those suitable to be used as scaffolds for tissue engineering applications. Scanning electron microscopy (SEM), bulk density, percentage of sample shrinkage and specific surface area measurements were used to highlight the sample morphological characteristics; whereas thermal gravimetric analysis (TGA) and differential scanning calorimetry (DSC) were performed to determine possible biopolymer modifications after processing. In addition, a solvent residue analysis was carried out to verify the proper performance of the supercritical processing.

## 2. Results and Discussion

### 2.1. Morphology of Agarose-Based Cryogels Obtained by a Fast-Cooling Step

Regarding the freeze-drying, the kind of porous structure of the final cryogel depends on the cooling rate [35,36]. According to the literature, the freezing rate affects the final sample morphology, since it influences the pore size after ice crystals sublimation. Espinoza et al. [37] studied the effect of this parameter on the cryogels obtained, demonstrating that a slow rate of freezing led to the formation of larger ice crystals and, consequently, to a larger expansion of pores in the structure. This phenomenon also led to the production of samples that were characterized by higher porosity values. Therefore, to have an idea of the influence of the freezing rate on the final scaffold morphology, in this work, two cooling rates were studied; i.e., 2.5 °C/min and 0.1 °C/min.

**Figure 1:**
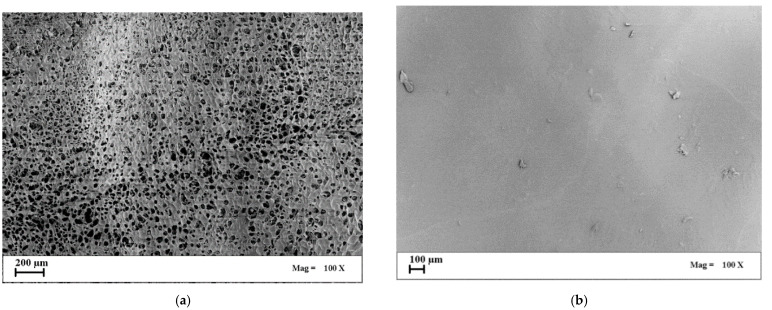
SEM images of cryogels surface obtained by a fast-cooling step: (**a**) 1 wt% agarose; (**b**) 8 wt% agarose.

In the first set of experiments, agarose cryogels were obtained by placing the native hydrogels in a cooling chamber set at −50 °C under vacuum, and the freezing step was performed in 30 min (fast cooling rate at 2.5 °C/min). The whole sublimation cycle was carried out overnight. As an example, the surfaces of the agarose-based cryogels produced at 1 wt% and 8 wt% of the biopolymer are shown in Figure 1.

A porous surface was obtained only in the case of the 1 wt% agarose-based cryogels (Figure 1a). At the larger polymer concentration (8 wt%), the surface of the samples was smooth and completely closed (Figure 1b). This result can be due to the increase in polymer concentration, which determined a major resistance to pore growth up to the sample surface.

**Figure 2:**
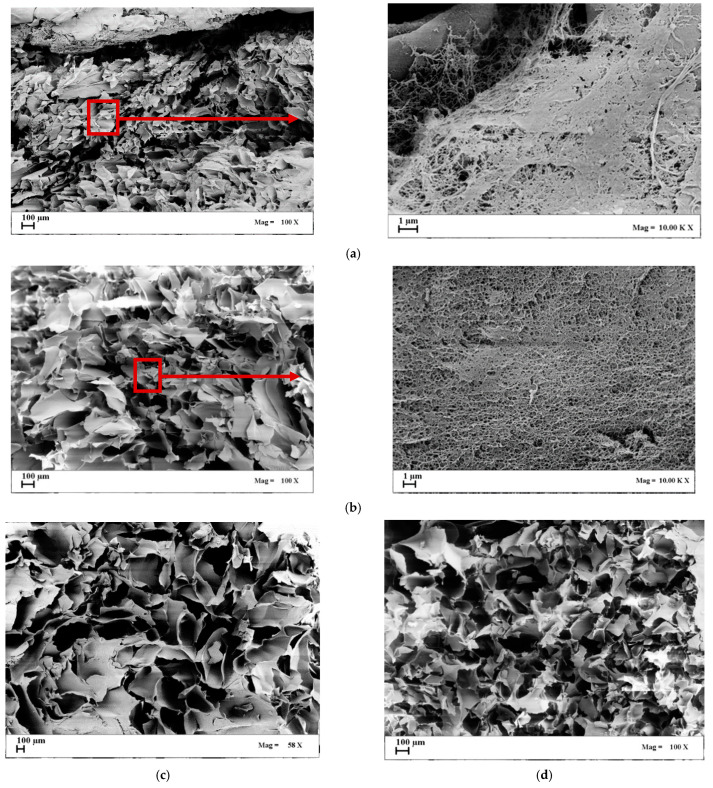
SEM images of cryogels section obtained by a fast-cooling step: (**a**) 1 wt% agarose; (**b**) 3 wt% agarose; (**c**) 5 wt% agarose; (**d**) 8 wt% agarose.

SEM images in Figure 2 show the sections of the cryogels, produced at the same cooling rate (2.5 °C/min). They were porous at all of the agarose concentrations tested: 1 wt%, 3 wt%, 5 wt% and 8 wt%.

These cryogels were characterized by pores that extended through the whole section of the samples; however, the pores showed an irregular shape. Overall, the produced cryogels showed large continuous interconnected pores (10–400 µm) with thin walls. Upon the increase in polymer concentration, the pores became more regular in shape, and the pore diameter approximately decreased from 480 ± 174 µm to 220 ± 95 µm, increasing the agarose concentration value from 5 wt% up to 8 wt%. At lower values of the biopolymer concentration (1 wt% and 3 wt%), a nanofibrous structure was also noted in some points of the section of agarose cryogels (see details in Figure 2a,b). At larger values of the concentration (5 wt% and 8 wt%), the nanofibrous structure was absent, and only macropores were present in the section (Figure 2c,d). Various authors found a correlation between the drying process and the drying rate: when faster cooling is carried out, smaller pores are produced that determine a slower drying rate, since a higher resistance to the water vapor flow, during sublimation, is provided [38,39,40,41].

This kind of macroporous structure could be used for cartilage tissue engineering applications, since it can support chondrocyte growth and matrix production upon implementation into a defect site. Moreover, macropores can promote the easy passing through of viscous fluids, such as blood, and the fast diffusion of nutrients, as well as the mass transport of nanoparticles and microparticles [42,43,44].

### 2.2. Morphology of Agarose-Based Cryogels Obtained by a Slow-Cooling Step

**Figure 3:**
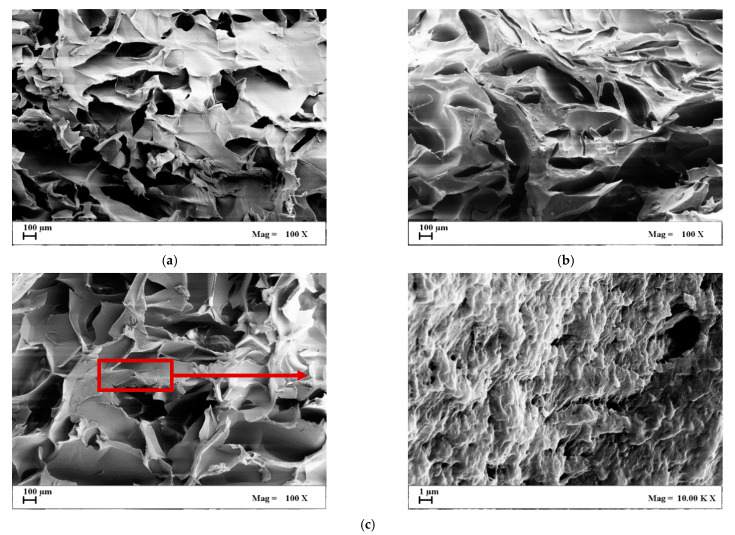
SEM images of cryogels section obtained by a slow-cooling step: (**a**) 3 wt% agarose; (**b**) 5 wt% agarose; (**c**) 8 wt% agarose.

In Figure 3, the section of cryogels that were produced using the same polymer amount of the previous samples, but at a freezing step of 12 h (slow cooling rate at 0.1 °C/min), is reported. Samples produced using 1 wt% of biopolymer concentration showed a severe shrinkage after processing (see Table 1) and, for this reason, are not reported.

According to the literature, when freezing occurs slowly, a lower number of nuclei is obtained and, subsequently, larger crystals are produced [36]. Since crystals form the pores after sublimation, the final cryogel microstructure will be characterized by larger average pore diameters. This effect can also be observed in this work, comparing Figure 2 with Figure 3.

Considering the morphology of the biopolymeric structures obtained at all of the investigated agarose concentrations shown in Figure 3, a competition between the nucleation rate of ice crystals and the rate of crystal growth can be hypothesized. In particular, the cross-sections reported in Figure 3 reveal a dense microstructure with randomly distributed irregular pores that could be due to a multiple orientation of crystal growth during freeze-drying. The nanofibrous structure observed in the samples of Figure 2 was not present in the cryogels produced using a slow cooling rate, and only a sort of rugosity appeared in some points of the cryogels section (see detail in Figure 3c).

The literature also reports that the different morphologies obtained by using two different cooling rates in the freeze-drying process can lead to a significant variation in Young’s modulus of the cryogels. Typically, cryogels characterized by larger pores and higher porosities generally show weaker mechanical properties and a significant drop in Young’s modulus [45,46].

Table 1 reports the results obtained for all of the cryogels produced in this work, in terms of: the percentage of sample shrinkage (after processing), bulk density and specific surface area. Comparing these values with the ones measured for the agarose-based aerogels produced via supercritical CO_2_ drying, it is possible to note that, in all cases, agarose cryogels suffered a larger percentage of sample shrinkage after processing. This result was consistent with the specific surface area measurements, which varied from 9 to 21 m^2^/g; i.e., these values were of an order of magnitude lower than the ones measured for agarose aerogels (specific surface area between 87 and 170 m^2^/g). The bulk density values were instead similar among agarose cryogels and aerogels.

### 2.3. Morphology of Agarose-Based Aerogels

**Figure 4:**
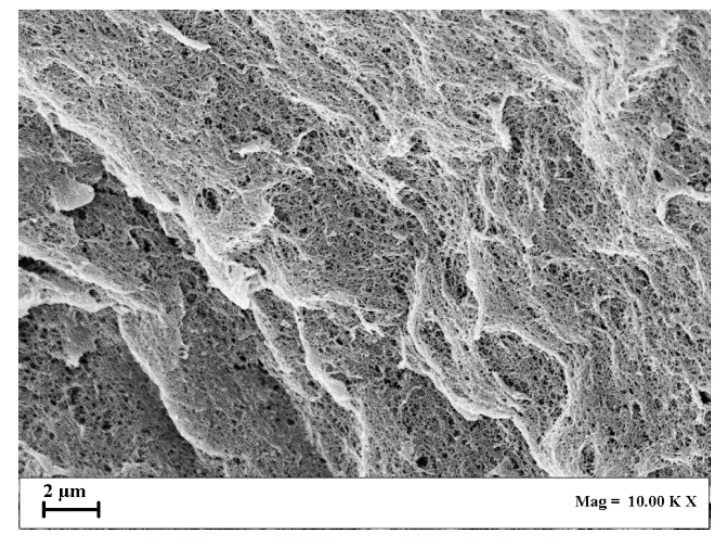
SEM image of the aerogel surface at 8 wt% agarose.

Agarose-based aerogels, prepared at increasing biopolymer concentration values, were produced by SC-CO_2_ drying, which was performed at 200 bar and 40 °C for 5 h. As an example, a SEM image of the surface of an 8 wt% agarose aerogel is reported in Figure 4.

Comparing Figure 4 with Figure 1b, it is possible to observe that SC-CO_2_ drying led to the production of a homogeneous nanoporous agarose structure, even in the case of the highest polymer concentration tested (8 wt%).

**Figure 5:**
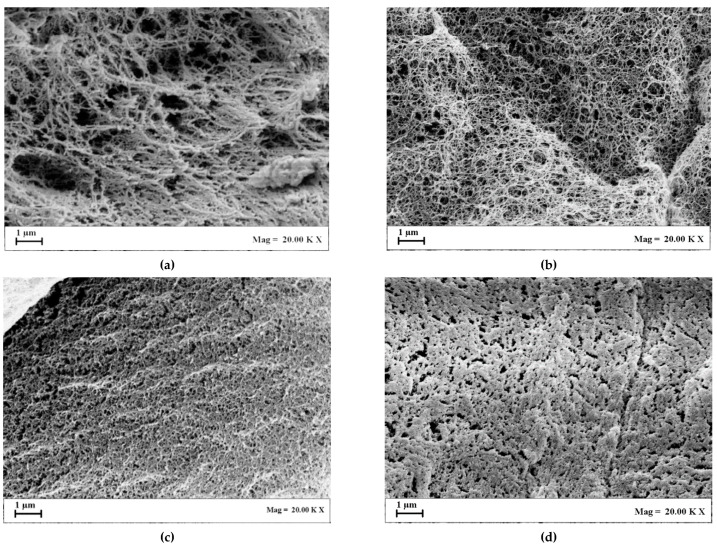
SEM images of aerogels section: (**a**) 1 wt% agarose; (**b**) 3 wt% agarose; (**c**) 5 wt% agarose; (**d**) 8 wt% agarose.

In Figure 5, SEM images of the section of agarose aerogels are reported.

Looking at Figure 5, a change in aerogel morphology can be observed at different biopolymer concentration values: i.e., it moved from nanofibrous to nanoporous. This behavior is the result of a gradual tendency of the pore size to decrease by increasing the polymer concentration [47,48]: using 1 wt% agarose, a more open and nanofibrous structure was obtained; whereas, using 8 wt% agarose, an almost closed and nanoporous structure was realized.

As reported in the Materials and Methods section, a multiple ethanol exchange was carried out before SC-CO_2_ drying, since water is not soluble in SC-CO_2_ at the ordinary pressures and temperatures adopted for this process. Therefore, water must be replaced in the hydrogel with a suitable solvent that is miscible in both water and CO_2_ (such as ethanol, obtaining an alcogel). SC-CO_2_ rapidly eliminates ethanol, transforming the alcogel into an aerogel and preserving the native biopolymer nanostructure, since the process is carried out at a negligible surface tension of the mixture ethanol + CO_2_. Specifically, CO_2_ acts as a zero-surface tension solvent and, when it diffuses in ethanol, the mixture also shows a near zero surface tension; i.e., a supercritical mixture is formed. Operating in this way, liquid cohesive forces are drastically reduced; this is the reason why the aerogel obtained by SC-CO_2_ drying preserves the original nanofibrous structure of the hydrogel/alcogel without observing structure collapse phenomena [3,30,31,33]. This result was also confirmed by the specific surface area obtained for these samples (see Table 1): indeed, it varied from 87 to 170 m^2^/g by increasing the agarose concentration in the starting hydrogel from 1 wt% to 8 wt%. Moreover, further evidence that the supercritical process was properly performed and that the water–ethanol exchange was correctly carried out was the percentage of samples shrinkage, summarized in Table 1, which was around 22% on average; i.e., much lower than the shrinkage measured for agarose cryogels.

A solvent residue analysis was carried out to verify if these aerogels were ethanol-free after the supercritical process; i.e., the aim was to ascertain if the drying process was successful. An ethanol residue less than 5 ppm was measured by GC-FID, confirming that the process conditions were properly selected.

Since solvent-free agarose aerogels, characterized by a morphology organized at nanoscale, like the tissue extracellular matrix [25,26,27], were successfully obtained by SC-CO_2_ drying, they can be considered good candidates as scaffolds for cell adhesion and growth [26,49,50] in tissue engineering.

### 2.4. DSC and TGA Results

**Figure 6:**
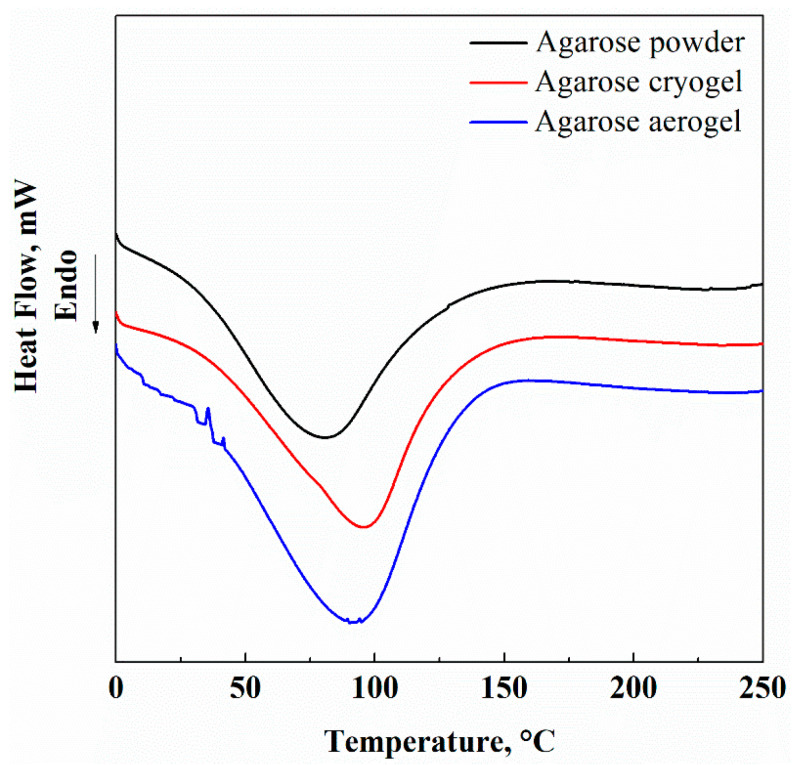
DSC of untreated agarose powder (black line), agarose cryogel (red line), agarose aerogel (blue line).

Thermograms of pure agarose, agarose-based cryogels and agarose-based aerogels are illustrated in Figure 6. This analysis was carried out to verify if the different processes influenced the thermal behavior of the porous agarose-based structures.

**Figure 7:**
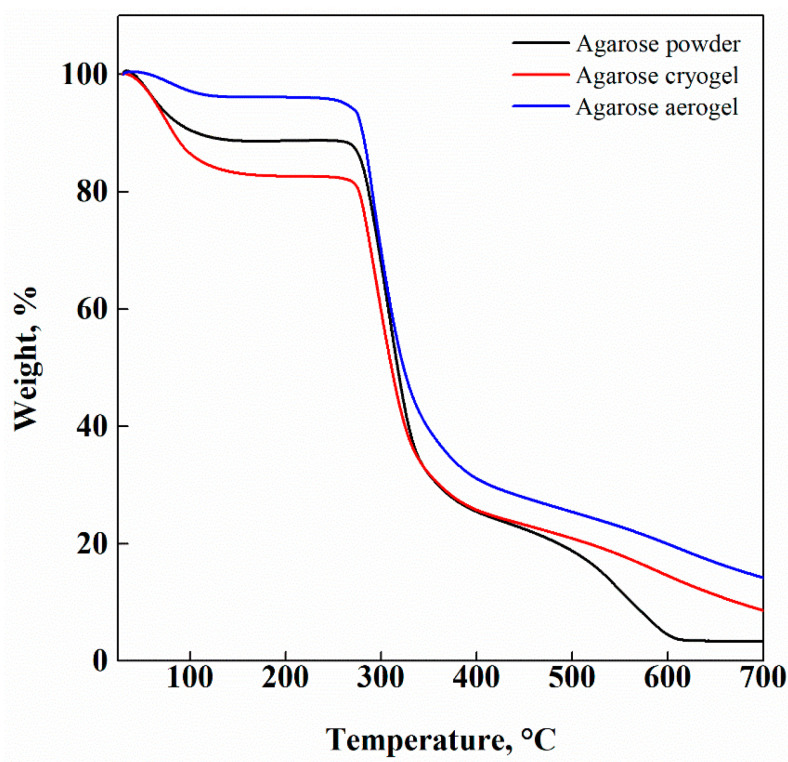
TGA of untreated agarose powder (black line), agarose cryogel (red line) and agarose aerogel (blue line).

DSC thermogram of untreated agarose powder exhibited a broad endothermic peak ranging from 50 °C to 120 °C. According to the literature, this peak is related to both the dehydration of the biopolymer, indicating the strong interaction between water molecules and agarose chains [51], and to the fusion of polymeric chains [52]. After the drying processes (freeze-drying and SC-CO_2_ drying), no thermal changes occurred in the samples. These results were also confirmed by the TGA reported in Figure 7.

TGA was carried out in the temperature range from 25 °C to 700 °C, under an inert atmosphere. The obtained curves evidenced that an overall weight loss of about 70% took place in the range between 300 °C and 350 °C. Moreover, the first weight loss, detected at 100 °C, indicated that physisorbed water was loosely bound at the agarose surface only in the case of the agarose powder and freeze-dried samples [53]; whereas the agarose-based aerogels did not show the peak related to water removal. This means that an enhanced thermal stability of the samples was achieved by SC-CO_2_ gel drying and that this process was efficiently performed.

## 3. Conclusions

Agarose cryogels and aerogels were successfully produced via freeze-drying and SC-CO_2_ drying, respectively. In general, the biopolymer concentration was the main parameter influencing the mean pore size and specific surface area of the samples. From a direct comparison among the morphology of agarose dried gels, it emerged that, in the case of freeze-drying, the cooling rate was the crucial parameter to control in order to obtain a homogeneous macroporous structure. SC-CO_2_ drying, instead, allowed for the preservation of the native nanoporous morphology of agarose-based gels, since solvent removal occurred at a negligible surface tension of the liquid solvent, which avoided the collapse of the delicate three-dimensional gel nanostructure.

## 4. Materials and Methods

### 4.1. Materials

Agarose (AG, Type I-A, low Electroendosmosis (EEO), powder) was supplied by Sigma Aldrich (Milan, Italy). CO_2_ (99.9% purity) was supplied by Morlando Group srl (Sant’Antimo, Naples, Italy).

### 4.2. Methods

**Figure 8:**
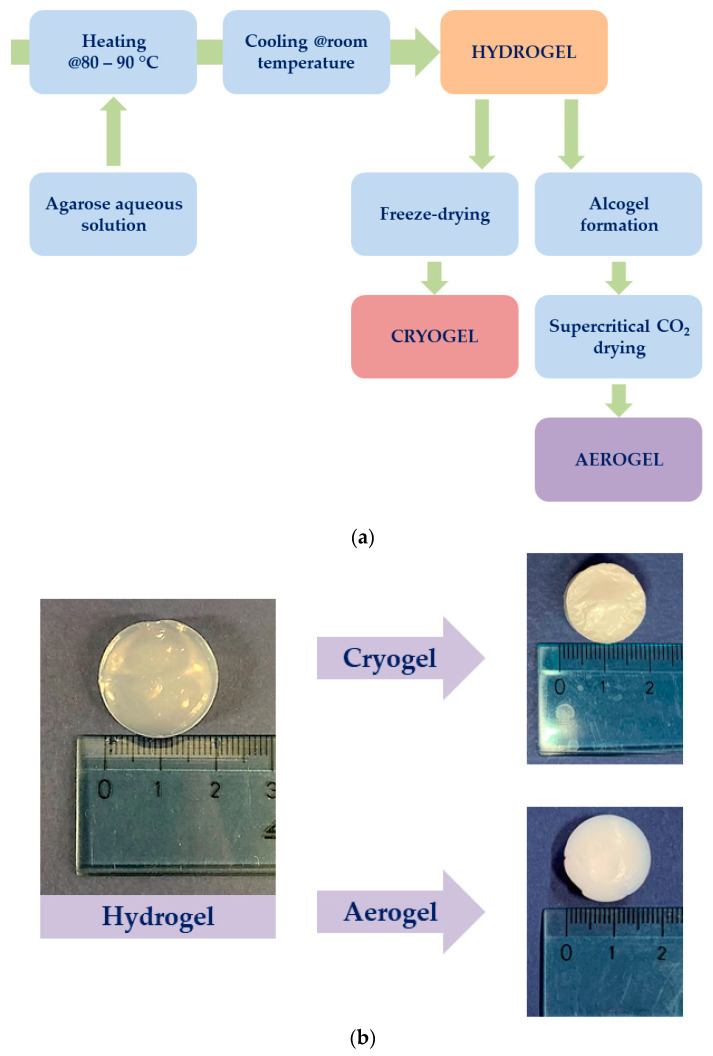
Schematic representation of agarose cryogels and aerogels preparation (**a**) and samples obtained (**b**).4.2.1. Hydrogel Preparation.

A schematic representation of the main steps involved in agarose cryogels and aerogels preparation is reported in Figure 8a; whereas Figure 8b shows the samples obtained.

#### 4.2.1. Hydrogel Preparation

Agarose hydrogels were formed starting from an aqueous solution heated up to 80–90 °C, which was subsequently cooled up to room temperature. Four different concentrations of agarose were investigated; i.e.,: 1 wt%, 3 wt%, 5 wt% and 8 wt%. After cooling, the agarose solutions resulted in a stable gel formation.

#### 4.2.2. Cryogel Preparation

Different amounts of agarose were solubilized in a volume of water required to reach 1 wt%, 3 wt%, 5 wt% and 8 wt% of biopolymer concentration. After hydrogels preparation, they were placed in the cooling chamber of a programmable cryostat (LyoQuest-55 Plus ECO, Seneco srl, Milan, Italy), set at −50 °C, under vacuum, overnight. Two different cooling steps were performed: the first one was conducted in 30 min and the second one was realized in 12 h. At the end of both processes, defrosting was carried out at 20 °C for 1 h.

#### 4.2.3. Aerogel Preparation

Agarose hydrogels were first transformed in alcogels using a multistep ethanol exchange. The solutions used for solvent exchange were prepared at increasing values of ethanol volume percentage (10, 30, 50, 70, 90, 100%), and each step lasted 1 h, except the last one (ethanol 100%), which lasted all night. Then, the alcogels were supercritically dried at 200 bar and 40 °C for 5 h. These operative conditions were previously optimized when testing natural polymer-based gels [25,30,54].

**Figure 9:**
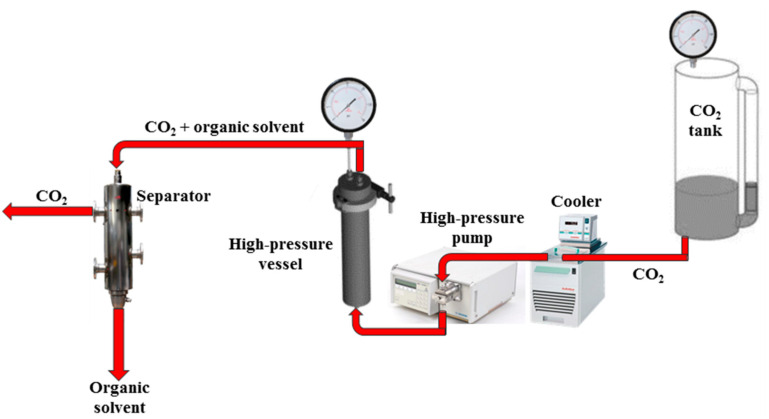
Schematic representation of the plant layout used for SC-CO_2_ drying.

The drying process was performed in a laboratory apparatus, shown in Figure 9, equipped with a 316 stainless steel high-pressure vessel with an internal volume of 200 mL. The alcogels were placed in the vessel, which was closed and filled from the bottom with SC-CO_2_ up to the desired pressure using a high-pressure pump (Gilson, mod. 305, Middleton, WI, USA). The apparatus operated in a continuous mode for 5 h. At the end, the vessel was slowly depressurized for 1 h. More details about the plant layout can be found in [55,56].

### 4.3. Characterizations

Agarose samples were cut using liquid nitrogen to preserve their inner structure and sputter coated with gold (Agar Auto Sputter Coater mod. 108 A, Stansted, UK) at 40 mA for 120 s; then, they were analyzed by a field emission scanning electron microscope (FESEM, mod. LEO 1525, Carl Zeiss SMT AG, Oberkochen, Germany) to observe the morphology. Sigma Scan and Microsoft Excel were used to evaluate the average pore size of agarose cryogels structure.

Differential scanning calorimetric (DSC) measurements were carried out using a Mettler Toledo DSC (822e Columbus, OH, USA) in a temperature range of 25–250 °C (heating rate of 10 °C/min), and using nitrogen as an inert gas, at a flow rate of 50 L/min [51].

Thermal gravimetric analysis (TGA) was carried out in a nitrogen atmosphere using a Mettler TGA/SDTA 851 (Columbus, OH, USA) thermal analyzer. The weight loss as a function of temperature was recorded by heating the sample from 25 °C to 700 °C, at a heating rate of 10 °C/min [57].

Ethanol residual content in the aerogels was measured by a headspace (HS) sampler (mod. 7694E, Hewlett Packard, Palo Alto, CA, USA) coupled with a gas chromatograph (GC) interfaced with a flame ionization detector (GC-FID, mod. 6890 GC-SYSTEM, Hewlett Packard, Palo Alto, CA, USA). Ethanol was separated using two fused-silica capillary columns that were connected in series by a press fit, with the first column (mod. Carbowax EASYSEP, Stepbios, Bologna, Italy) connected to the detector (30 m length, 0.53 mm ID, 1 μm film thickness), and the second one (mod. Cp Sil 5CB CHROMPACK, Stepbios, Bologna, Italy) connected to the injector (25 m length, 0.53 mm ID, 5 μm film thickness). GC conditions were reported in the USP 467, with some modifications: the oven temperature was raised from 45 °C to 210 °C for 15 min, the injector was maintained at 135 °C (split mode, ratio 4:1), and helium was used as the carrier gas (5 mL/min). Headspace conditions were the following ones: equilibration time, 30 min at 95 °C; pressurization time, 0.15 min; and loop fill time, 0.15 min. Headspace samples were prepared in 20 mL vials filled with 3 mL of an internal standard (1,2-dimethylimidazole) and 500 mg of NaCl and water (0.75 mL), in which, part of agarose aerogels was suspended [56].

Bulk density of agarose cryogels and aerogels was measured by the ratio between sample mass and volume, expressed in g/cm^3^.

The percentage of samples shrinkage was measured using the following equation: $\frac{\left(V_{i}-V_{f}\right)}{V_{i}}\cdot 100$, where *V_i_* and *V_f_* are the agarose hydrogel volume and the final cryo/aerogel volume, respectively.

Brunauer–Emmett–Teller (BET) specific surface area of the samples was determined by a Nova 1200e Surface Area (Quantachrome Instruments, Boynton Beach, FL, USA). A total of 200 mg of agarose sample was analyzed by N_2_ adsorption at −196 °C.

## Figures and Tables

**Table 1:** Percentage of shrinkage, bulk density and specific surface area measurements of the agarose cryogels and aerogels produced in this work.

**Freeze-Drying,** **Fast Cooling Rate**	**Sample Shrinkage, %**	**Bulk Density, g/cm^3^**	**Specific Surface Area, m^2^/g**
1 wt% agarose cryogel	98	0.54	9
3 wt% agarose cryogel	52	0.06	13
5 wt% agarose cryogel	28	0.11	13
8 wt% agarose cryogel	28	0.15	14
**Freeze-Drying,** **Slow Cooling Rate**	**Sample Shrinkage, %**	**Bulk Density, g/cm^3^**	**Specific Surface Area, m^2^/g**
1 wt% agarose cryogel	68	0.03	10
3 wt% agarose cryogel	36	0.05	11
5 wt% agarose cryogel	24	0.07	13
8 wt% agarose cryogel	24	0.14	21
**SC-CO_2_ Drying**	**Sample Shrinkage, %**	**Bulk Density, g/cm^3^**	**Specific Surface Area, m^2^/g**
1 wt% agarose aerogel	28	0.02	87
3 wt% agarose aerogel	20	0.02	154
5 wt% agarose aerogel	21	0.07	156
8 wt% agarose aerogel	21	0.13	170
